# Stage IV gastric cancer with microsatellite instability–high achieving long-term survival by gastrectomy after nivolumab as third-line therapy: a case report and literature review

**DOI:** 10.1186/s40792-024-02022-5

**Published:** 2024-09-19

**Authors:** Hideki Kumagai, Shigeaki Baba, Haruka Nikai, Ryosuke Fujisawa, Misato Shimooki, Akira Sasaki

**Affiliations:** Department of Surgery, Iwate Medical University, 2-1-1 Idaidori, Yahaba, Iwate, 028-3695 Japan

**Keywords:** Gastric cancer, Gastrectomy, Nivolumab, Microsatellite instability

## Abstract

**Background:**

The prognosis for stage IV gastric cancer remains poor; however, the advent of immune checkpoint inhibitors (ICIs) such as nivolumab has increased the number of patients with long-term survival. Patients with microsatellite instability (MSI)–high gastric cancer have been recognized as a highly effective population for ICIs. Herein, we report a patient with MSI–high advanced gastric cancer treated with gastrectomy after the administration of nivolumab as third-line therapy.

**Case presentation:**

A 73-year-old woman presented with a type 3 tumor in the lower part of the gastric body, which was diagnosed as gastric cancer through biopsy. Staging laparoscopy revealed that the tumor had invaded the pancreas and the posterior lobe of the transverse mesocolon, and disseminated nodules were found near the ligament of Treitz. After 4 courses of S-1 plus cisplatin therapy, laparoscopic gastrojejunal bypass was performed because of difficulty in oral intake. She received S-1 plus oxaliplatin therapy after a gastrojejunal bypass; however, her regional lymph nodes were enlarged. After six courses of paclitaxel plus ramucirumab as second-line chemotherapy, computed tomography (CT) showed exacerbation of peritoneal dissemination; thus, nivolumab was selected as the third-line therapy. The tumor was characterized by MSI–high. At 24 courses, CT and gastroscopy revealed a complete clinical response of the tumor; however, re-growth of the primary tumor was observed at 36 courses. The patient underwent distal gastrectomy with D1 + lymph node dissection, and received S-1 monotherapy as adjuvant therapy for 1 year. No recurrence was noted at 39 months after the surgery.

**Conclusions:**

We report a patient with highly advanced gastric cancer with peritoneal dissemination, which worsened during second-line therapy and was successfully treated with gastrectomy after nivolumab administration as a third-line therapy. MSI–high gastric cancer is a target that should be actively considered for the administration of ICIs, such as nivolumab, and multidisciplinary treatment combined with chemotherapy and gastrectomy, including conversion surgery, can lead to patients’ long-term survival.

## Background

The prognosis for stage IV gastric cancer remains severe, with median overall survival (OS) generally around 6–17 months [1, 2]. However, based on the results of the Attraction-2 [3] and Checkmate-649 trials [4], nivolumab has been accepted as the standard treatment for unresectable gastric cancer, and the number of patients achieving long-term survival is increasing. Although there are few reports on conversion surgery (CS) after nivolumab administration, an increase in CS rates is expected in the future with the spread of immune checkpoint inhibitors (ICIs), such as nivolumab. The accumulation of clinical experience in achieving long-term survival in patients with surgery can provide useful information for developing treatment strategies for highly advanced gastric cancer.

Microsatellite instability (MSI) is a biomarker in solid tumors, such as gastric cancer, and the proportion of MSI–high in patients with gastric cancer is approximately 3–7% [4, 5]. In a subgroup analysis of Checkmate-649 trial, the hazard ratio (HR) for OS with nivolumab plus chemotherapy relative to chemotherapy in all patients with MSI–high gastric cancer was 0.37 (95% CI [0.16–0.87]) [4]. In other words, MSI–high gastric cancer should be actively considered for the administration of ICIs, such as nivolumab.

Herein, we report a patient with stage IV MSI–high gastric cancer with peritoneal dissemination, which worsened with second-line therapy and was successfully treated with distal gastrectomy and lymph node dissection after nivolumab as a third-line chemotherapy and achieved long-term survival.

## Case presentation

**Fig. 1:**
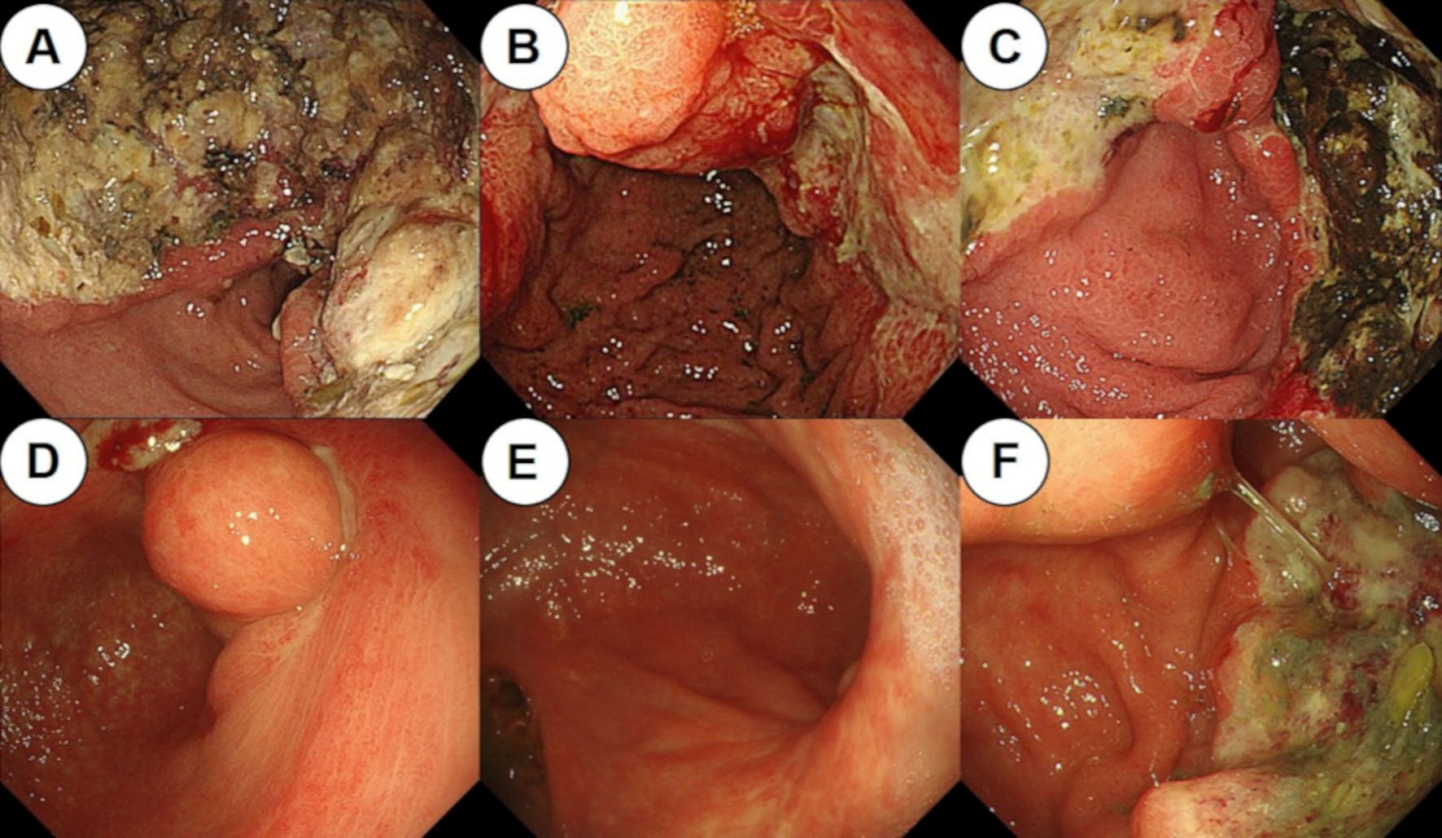
Change in esophagogastroduodenoscopy (EGD) findings. EGD showed the primary tumor in the lower part of the gastric body before chemotherapy (**A**), before laparoscopic gastrojejunostomy (**B**), before nivolumab administration as the third-line therapy (**C**), at 16 courses of nivolumab therapy (**D**), at 24 courses of nivolumab therapy (**E**), and after 36 courses of nivolumab therapy (**F**)

**Fig. 2:**
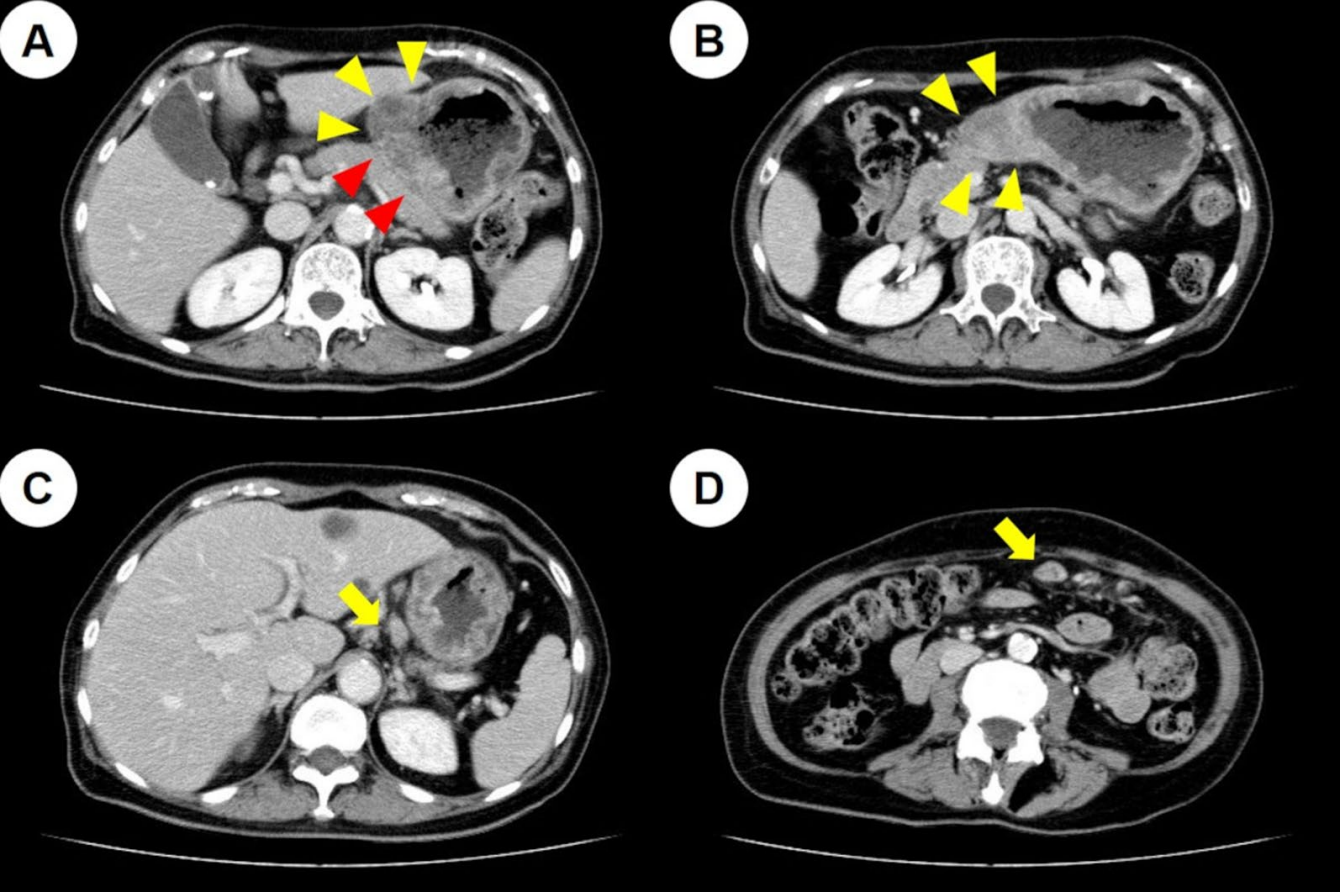
Computed tomography findings before chemotherapy. **A** Wall thickening in the lower part of the gastric body (yellow arrowheads) and unclear boundaries between the tumor and the pancreas (red arrowheads) were demonstrated. **B** Full-circumferential wall thickening in the lower part of the gastric body was observed (yellow arrowheads). **C** Enlarged lymph node of lesser curvature was observed (yellow arrow). **D** Enlarged lymph node of greater curvature was observed (yellow arrow)

**Fig. 3:**
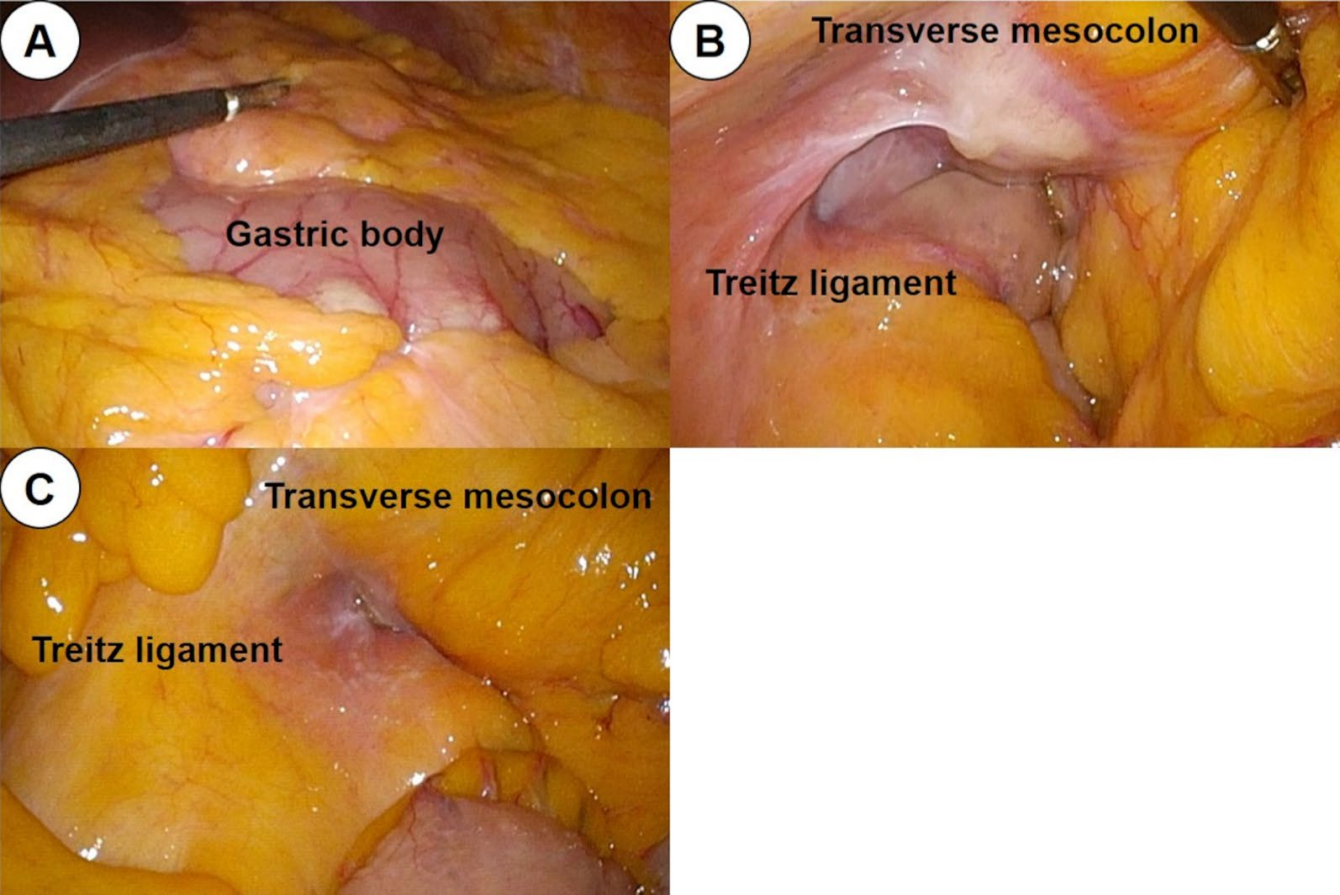
Intraoperative findings of staging laparoscopy. **A** Tumor was localized in the lower part of the gastric body and exposed on the serous surface. **B** Tumor invaded the posterior lobe of the transverse mesocolon. **C** White nodules suggesting peritoneal dissemination were found near the Treitz ligament

A 73-year-old woman presented at our hospital with shortness of breath and fatigue. Blood examination revealed anemia with an Hb of 6.5 g/dL, and esophagogastroduodenoscopy (EGD) revealed a type 3 tumor in the lower part of the stomach body (Fig. 1A). The patient was diagnosed with gastric cancer by pathological examination of the biopsied specimen, and was referred to our hospital for treatment. She had a medical history of hypertension and dyslipidemia, no allergies, while her mother had a history of pancreatic cancer. Tumor markers, including carcinoembryonic antigen, carbohydrate antigen 19–9, and carbohydrate antigen 125, were within normal ranges. Computed tomography (CT) revealed full circumferential wall thickening in the lower part of the gastric body, while the boundary with the pancreas was unclear (Fig. 2A, B). In addition, enlarged lymph nodes in the lesser and greater curvatures were observed, suggesting metastasis (Fig. 2C, D). Staging laparoscopy revealed a tumor located in the lower part of the gastric body (Fig. 3A), which had invaded the pancreas and posterior lobe of the transverse mesocolon (Fig. 3B), and white nodules were found near the ligament of Treitz (Fig. 3C). Intraoperative frozen sections using a specimen of white nodules and peritoneal washing cytology revealed no evidence of malignancy. However, the above macroscopic intraoperative findings suggested the presence of peritoneal dissemination. Based on these findings, the patient was finally diagnosed with unresectable advanced gastric cancer (cT4b [pancreas and posterior lobe of the transverse mesocolon] N3 M1 [P1a] Stage IVB) [6]. The human epidermal growth factor receptor 2 status was negative, and S-1 plus cisplatin (SP) therapy (S-1, 100 mg/day; cisplatin, 60 mg/m^2^) was selected as the first-line treatment. Because she had neutropenia (grade 4) during the first course, the dose was reduced to S-1 (80 mg/day) and CDDP: 45 mg/m^2^/day from the second course. After 4 courses of SP therapy, she experienced difficulty with oral intake. CT revealed no significant changes in the primary tumor; however, the regional lymph nodes were shrinking. EGD demonstrated flattening of the tumor margin, indicating a therapeutic effect, while the tumor stenosis worsened (Fig. 1B). The patient underwent laparoscopic gastrojejunostomy with Billroth II and Braun anastomoses and tolerated oral intake.

**Fig. 4:**
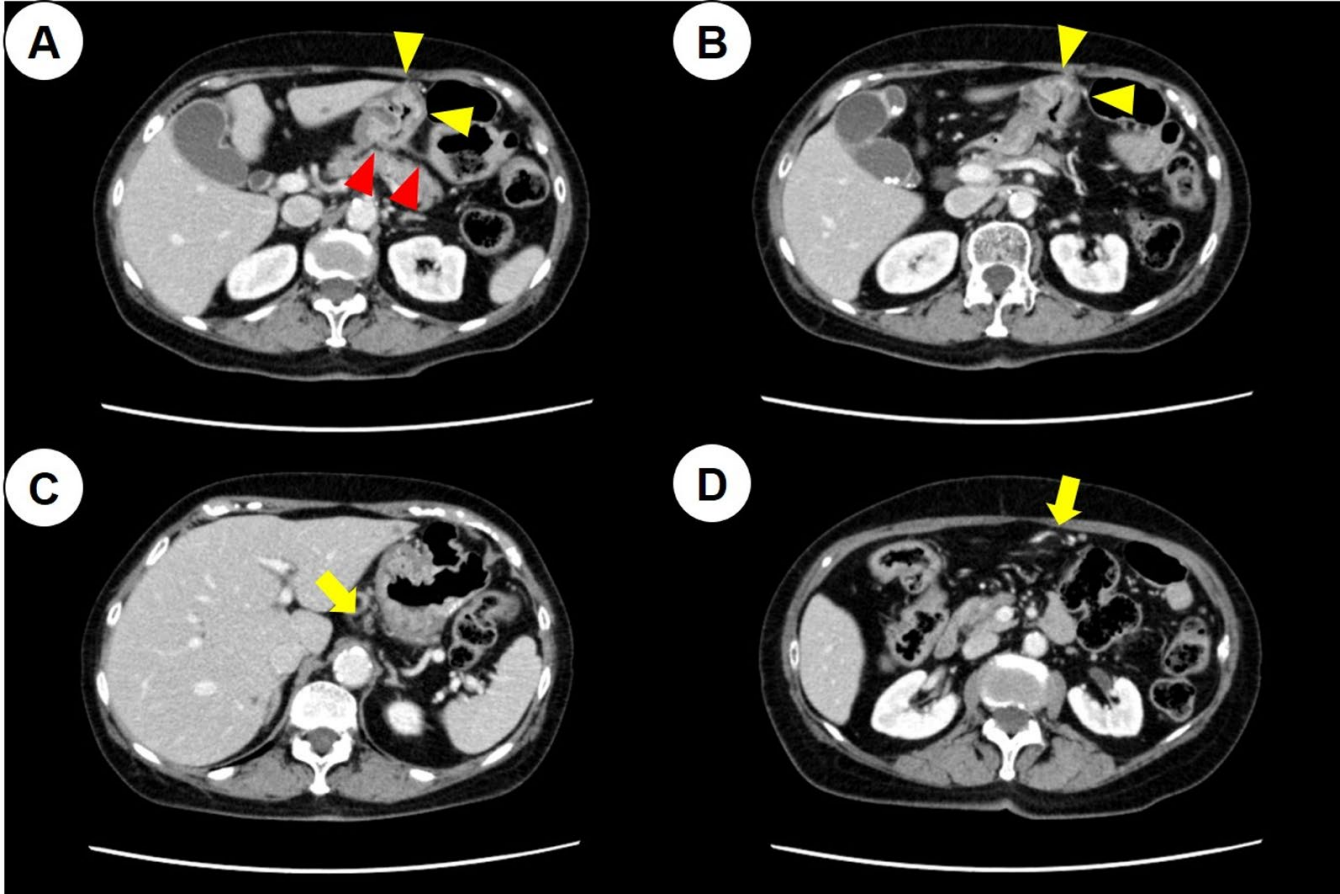
Computed tomography (CT) findings before gastrectomy. **A**, **B** CT showed that the primary lesion had regressed compared to before chemotherapy (yellow arrowheads), and the boundary between the tumor and the pancreas was clear (red arrowheads). **C**, **D** CT showed that the regional lymph nodes in lesser and greater curvatures had also shrunk (yellow arrows)

**Fig. 5:**
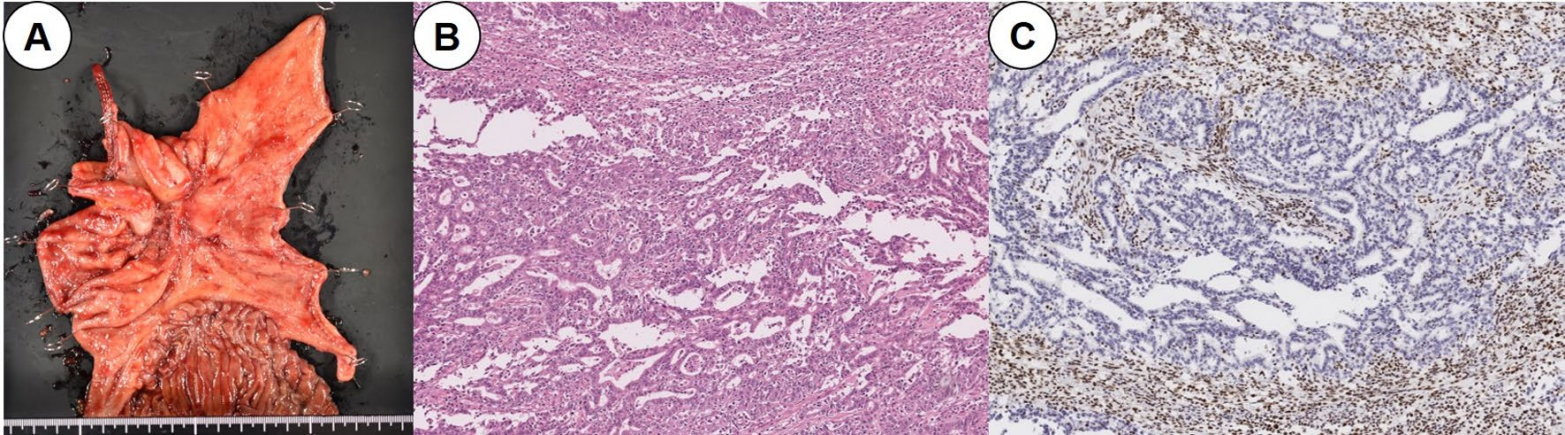
Macroscopic and histopathological findings. **A** Macroscopic findings revealed a type 3 tumor located in the lower part of the stomach body. **B** Histopathological findings showed a moderately differentiated adenocarcinoma with tumor-infiltrating lymphocytes. The histological effect of chemotherapy was grade 1a. **C** Immunohistochemical analysis showed that tumor cells had lost the expression of mutL homolog 1

**Fig. 6:**
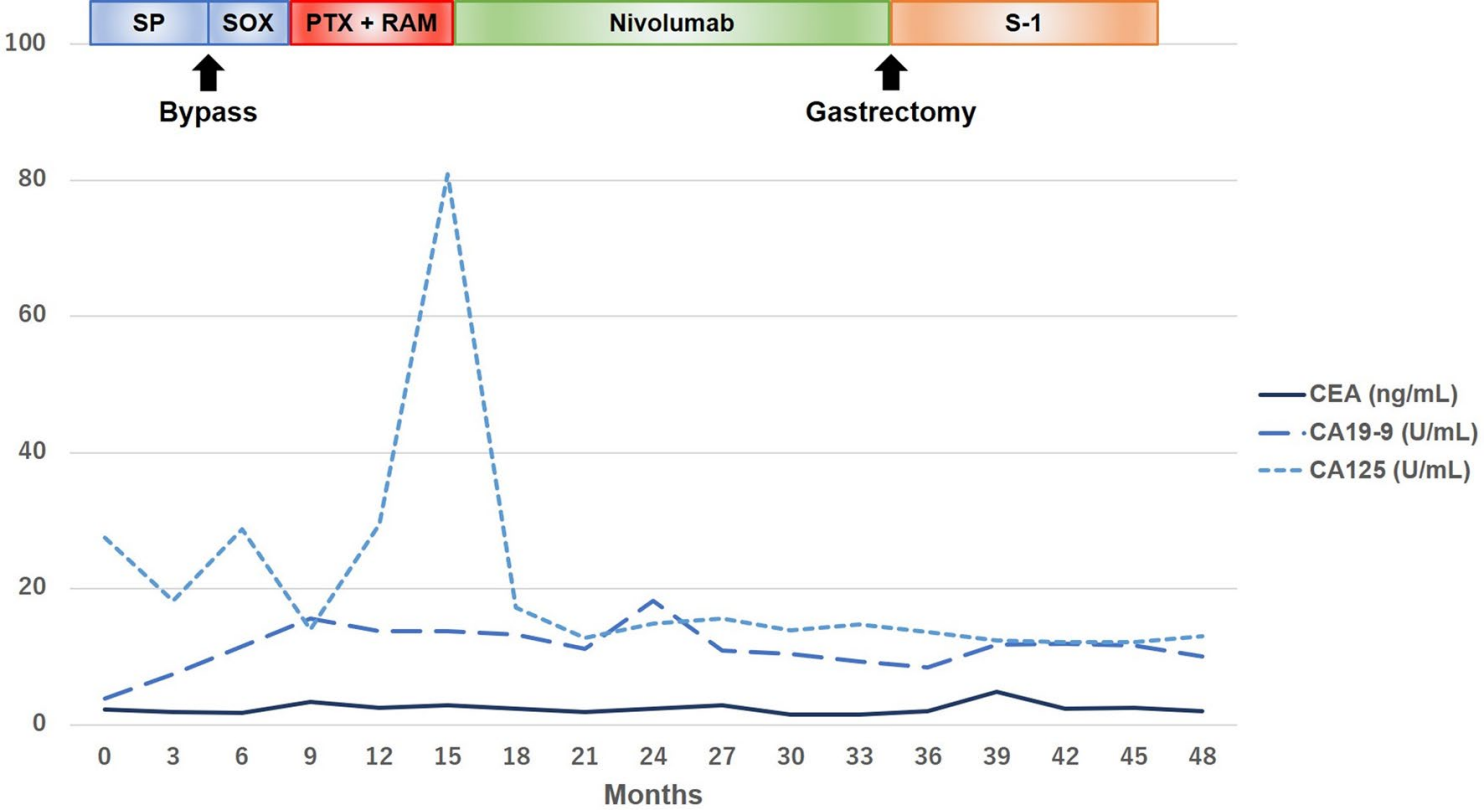
Patient timeline. An increase in CA125 was observed at the timing of switching from PTX + RAM to nivolumab therapy. SP, S-1 plus cisplatin; SOX, S-1 plus oxaliplatin; PTX, paclitaxel; RAM, ramucirumab; CEA, carcinoembryonic antigen; CA19-9, carbohydrate antigen 19-9; CA125, carbohydrate antigen 125

Considering the deterioration of renal function and the risk of neurological problems due to cisplatin, chemotherapy was resumed after switching to S-1 plus oxaliplatin (SOX) therapy (S-1,80 mg/day; oxaliplatin, 80 mg/m^2^/day) 1 month after surgery. After four courses of SOX therapy, an increase in the regional lymph nodes was observed, and the patient was switched to weekly paclitaxel (PTX) plus ramucirumab (RAM) therapy (PTX: 80 mg/m^2^, RAM: 8 mg/kg) as a second-line therapy. After six courses of PTX + RAM therapy, CT showed an increase in ascites and the CT value of the omentum, which suggested an exacerbation of peritoneal dissemination; nivolumab therapy (240 mg/body) was then initiated as the third-line treatment. Additional histopathological examination after the third-line treatment revealed that the gastric cancer was characterized by MSI–high status and a programmed cell death ligand 1 (PD-L1) combined positive score (CPS) of ≥ 5. After 6 courses, hypothyroidism was observed, and thyroxine replacement therapy was initiated. EGD after 16 courses of nivolumab therapy revealed that the primary lesion had shrunk dramatically (Fig. 1D), and after 24 courses, CT and EGD revealed a clinical complete response of the tumor (Fig. 1E). However, after 36 courses, regrowth of the primary lesion was observed (Fig. 1F). CT showed that the boundary between the primary lesion and the pancreas was clear and that the regional lymph node had been shrinking (Fig. 4). Therefore, distal gastrectomy and lymph node dissection, primarily aimed at preventing bleeding and stenosis associated with tumor regrowth, was performed. First, diagnostic laparoscopy was performed, which revealed no obvious peritoneal dissemination. Ascites cytology also revealed no malignancy; thus, laparotomy was performed through a midline epigastric incision. The primary tumor was mainly localized in the angulus, and shortening of the lesser curvature was observed. There was no invasion of the pancreas; however, the invasion of the posterior lobe of the transverse mesocolon persisted, and the mesocolon was resected. The jejunum, duodenum, and upper stomach were dissected using a linear stapler. Distal gastrectomy with Roux-en-Y reconstruction and D1 + lymph node dissection were performed. The final histopathological diagnosis was ypT4bN0M0 Stage IIIA [6]. Macroscopic findings revealed a type 3 tumor in the lower part of the stomach body (Fig. 5A), and histopathological findings showed that the tumor was a moderately differentiated adenocarcinoma (Fig. 5B). In addition, immunohistochemical analysis indicated that the tumor cells had lost the expression of mutL homolog 1 (Fig. 5C). The histological effect of the chemotherapy was grade 1a. Considering tumor growth during nivolumab therapy, S-1 monotherapy (80 mg/day) was selected as adjuvant chemotherapy and continued for 1 year after surgery. Six years have passed since the first visit to our hospital and 3 years have passed since the last surgery; however, no recurrence has been observed without chemotherapy. Figure 6 shows the patient’s clinical course and changes in tumor markers.

## Discussion

In this study, we report the long-term survival of a patient after third-line nivolumab therapy for advanced gastric cancer with peritoneal dissemination, which worsened after second-line therapy. To the best of our knowledge, this patient had the longest follow-up and survival duration among patients who underwent gastrectomy after third-line nivolumab therapy.

The results of the Attraction-2 trial were reported in 2018, and the usefulness of nivolumab therapy as a third-line chemotherapy for gastric cancer was recognized [3]. In addition, the results of the CheckMate-649 trial indicated the usefulness of adding nivolumab to chemotherapy in previously untreated patients with advanced gastric cancer [4]. As a result of these findings, nivolumab is currently the standard treatment for gastric cancer worldwide. One of the response characteristics of nivolumab is that there are a certain number of patients who can achieve long-term survival, which is called the “tail” of the Kaplan–Meier curve [7]. In the CheckMate-649 trial, the 3-year OS of patients treated with nivolumab plus chemotherapy was approximately 20% [4], and further long-term follow-up results are expected. In addition, in a subgroup analysis of the trial, the HR of nivolumab to OS for MSI–high gastric cancer was approximately 0.3 [4]; thus, MSI–high gastric cancer is a target for which the administration of ICIs, such as nivolumab, should be actively considered. Hidaka et al. reported on patient for whom CS was performed after six courses of pembrolizumab for MSI–high unresectable advanced gastric cancer, and a pathological complete response was confirmed [8]. Our patient had an unresectable, highly advanced gastric cancer with MSI–high, but was successfully treated with distal gastrectomy and lymph node dissection after nivolumab therapy as the third-line treatment. Even if gastric cancer is initially advanced and unresectable, a multidisciplinary treatment strategy with a view to radical surgery, including CS, can be devised with the administration of ICIs, such as nivolumab and pembrolizumab, in patients with MSI–high gastric cancers.

The most prominent factor in the patient's long-term survival was that she had MSI–high gastric cancer, and the third-line nivolumab therapy had a dramatic effect. However, it is also important to note that the patient underwent a bypass procedure and continued chemotherapy, even though oral intake was difficult at the first-line treatment, and that there were no immune-related adverse events that required discontinuation of nivolumab. As mentioned above, for advanced, unresectable gastric cancer, it is important to develop a multidisciplinary treatment strategy that does not overlook the opportunities for radical surgery.

We searched PubMed and MEDLINE for keywords such as gastric cancer, nivolumab, and conversion surgery and found 11 reports [9–19] in which gastrectomy for highly advanced gastric cancer was performed after nivolumab therapy (Table 1). Ten patients, including our patient, underwent gastrectomy after third-line nivolumab therapy, and two patients underwent gastrectomy after first-line nivolumab therapy. The median age was 72 years (range: 65–80), and the male-to-female ratio was 9:3. The most common tumor site was the gastric body, and the unresectable factors were liver metastases in four, peritoneal dissemination in four, and para-aortic lymph node metastases in three patients. MSI–high gastric cancer was observed only in our patient. The median course of nivolumab therapy in the third-line was 21.5 courses (7–36), and our patient had the highest number of courses of nivolumab therapy. The surgical procedure was the resection of primary lesion and distant metastases in patients with liver and para-aortic lymph node metastases. Resection of the primary lesion and regional lymph nodes was performed in patients with peritoneal dissemination, including in our patient. As an adjuvant therapy after CS, which remains controversial [20], nivolumab therapy tended to be selected for patients who responded to nivolumab before gastrectomy. Our patient received S-1 monotherapy as adjuvant therapy because the primary lesion enlarged during nivolumab therapy before gastrectomy. The median follow-up period was 9 months (3–39), and one patient died from the recurrence of gastric cancer. Our patient had the longest follow-up and survival periods.

**Table 1:** Overview of patients who were treated with gastrectomy after nivolumab therapy

Case	First author	Age	Gender	Tumor location	Unresectable factor	Biomarker	Chemotherapy before gastrectomy	Surgical procedure	Adjuvant therapy	Follow-up duration after gastrectomy	Outcome	
1	Toyota S [9]	75	Male	Antrum	Peritoneal dissemination	Unknown	SOX 2 courses PTX + RAM 7 courses Nivolumab 23 courses	Open distal gastrectomy D1 + dissection	None	7 months	Alive without recurrence	
2	Matsumoto R [10]	68	Female	Body	Liver	HER2 negative	SOX 6 courses PTX + RAM 6 courses Nivolumab 20 courses	Laparoscopic distal gastrectomy D2 dissection	Nivolumab	3 months	Alive without recurrence	
3	Lin CP [11]	65	Male	Antrum	Liver	Unknown	FLP 3 courses PTX + RAM Nivolumab 14 courses	Open distal gastrectomy D2 dissection Left lateral liver segmentectomy	Nivolumab	28 months	Alive without recurrence	
4	Toyota S [12]	70	Male	Body	Para-aortic lymph nodes	HER2 negative	SOX 3 courses PTX + RAM 7 courses Nivolumab 24 courses	Open total gastrectomy D2 dissection Distal pancreatosplenectomy Partial transverse colectomy	None	7 months	Alive without recurrence	
5	Kumamoto T [13]	65	Male	Esophagogastric junction	Para-aortic lymph nodes	HER2 negative Microsatellite stable PD-L1 CPS 1–5	SOX 5 courses Nab-PTX + RAM 5 courses Nivolumab 8 courses	Laparoscopic Ivor-Lewis oesophagectomy Abdominal D2 dissection Transhiatal lymph node dissection	Unknown	3 months	Alive without recurrence	
6	Toyota Y [14]	69	Male	Cardia and Body	Peritoneal dissemination	HER2 negative Microsatellite stable PD-L1 CPS > 5	SOX 14 courses Nab-PTX + RAM 2 courses Nivolumab 12 courses	Open total gastrectomy D2 dissection Paraaortic lymphadenectomy	Nivolumab	12 months	Alive without recurrence	
7	Watanabe H [15]	73	Male	Upper body	Liver	HER2 positive	XP + Tmab 12 courses PTX + RAM 25 courses Nivolumab 31 courses	Open total gastrectomy D2 dissection	Unknown	9 months	Alive without recurrence	
8	Ushimaru Y [16]	74	Male	Middle to Lower body	Para-aortic lymph nodes	HER2 positive PD-L1 positive	XP + Tmab 10 courses PTX + RAM 5 courses Nivolumab 27 courses	Laparoscopic distal gastrectomy D2 dissection	Nivolumab	14 months	Alive without recurrence	
9	Sato S [17]	72	Male	Middle to Lower body	Liver	HER2 negative Microsatellite stable	SOX 4 courses S-1 7 courses PTX + RAM 6 courses Nivolumab 7 courses	First: Open total gastrectomy D2 dissection Left lateral liver segmentectomy Partial hepatectomy (segments 6 and 7) Second: Partial hepatectomy (segments 4 and 5)	Nivolumab	Unknown (60 months after first-line chemotherapy)	Death of gastric cancer	
10	Kawaguchi D [18]	80	Male	Lower body to pylorus	Positive ascites cytology Bulky lymph node	PD-L1 CPS > 5	SOX + Nivolumab 7 courses	Unknown	None	18 months	Alive without recurrence	
11	Noma T [19]	60 s	Female	Body	Peritoneal dissemination	HER2 negative	SOX + Nivolumab 10 courses	Laparoscopic total gastrectomy D2 dissection Resection of the round ligament of the liver	S-1	7 months	Alive without recurrence	
12	Kumagai H	73	Female	Body	Peritoneal dissemination	HER2 negative MSI–high	SP 4 courses SOX 4 courses PTX + RAM 6 courses Nivolumab 36 courses	Open distal gastrectomy D1 + dissection	S-1 (12 months)	39 months	Alive without recurrence	

*HER2* human epidermal growth factor receptor 2, *PD-L1* programmed death ligand 1, *CPS* combined positive score, *MSI* microsatellite instability, *CS* conversion surgery, *SOX* S-1 plus oxaliplatin, *PTX* paclitaxel, *RAM* ramucirumab, *FLP* 5-fluorouracil, folinic acid, and cisplatin, *Nab-PTX* nab-paclitaxel, *XP* capecitabine and cisplatin, *Tmab* trastuzumab, *SP* S-1 and cisplatin

## Conclusions

In this study, we report a patient who had highly advanced gastric cancer with peritoneal dissemination, which worsened with second-line therapy. The patient was successfully treated with distal gastrectomy and lymph node dissection after nivolumab administration as third-line therapy, and achieved long-term survival. MSI–high gastric cancer is a target that should be actively considered for the administration of ICIs, such as nivolumab, and multidisciplinary treatment combined with chemotherapy and gastrectomy, including CS, can lead to long-term survival in such patients.

## Data Availability

Not applicable.

## Abbreviations

OS: Overall survival
CS: Conversion surgery
ICIs: Immune checkpoint inhibitors
MSI: Microsatellite instability
HR: Hazard ratio
EGD: Esophagogastroduodenoscopy
CT: Computed tomography
SP: S-1 plus cisplatin
SOX: S-1 plus oxaliplatin
PTX: Paclitaxel
RAM: Ramucirumab
PD-L1: Programmed cell death ligand 1
CPS: Combined positive score
